# Ecological concrete by partially substitution of cement with Cameroonian corn stover ash

**DOI:** 10.1016/j.heliyon.2024.e41424

**Published:** 2024-12-24

**Authors:** Martial Ngnihamye Nde, Seidou Youssoufa, Fabien Kenmogne, Merveille Meto Kuigne, Michel Mbessa, Emmanuel Yamb Bell, Didier Fokwa

**Affiliations:** a Department of Civil Engineering, National Advanced School of Public Works, P.O. Box 510, Yaoundé, Cameroon; b Mechanics Laboratory, Doctoral Training Unit in Engineering Sciences, Doctoral School of Fundamental and Applied Sciences, University of Douala, P.O. Box: 2701, Douala, Cameroon; c Laboratory of Research in Mechanics and Materials, National High School Polytechnic Douala, University of Douala, P.O. Box: 2701, Douala, Cameroon; d Department of Civil Engineering, Advanced Teachers Training College of the Technical Education, P.O. Box 1872, University of Douala, Cameroon

**Keywords:** Ecological material, Corn stover ash, Concrete, Compressive strength, Agricultural waste management

## Abstract

This study focuses on the influence of the partial substitution of cement by Cameroonian corn stover ash (CCSA) on the physical and mechanical behavior of concrete. For this, as materials used, one has first the corn stovers coming from the Bandjoun town in the Koung-khi division, in the West region of Cameroon, which are used to obtain the ashes, while the sand used, came from the Sanaga River in the coastal region of Cameroon. In order to obtain the CCSA, the corn stover is calcined in an oven at 600 °C for 6 h and then characterized; the characterization included infrared spectrometry, X-ray fluorescence spectrometry, fineness of grinding, and absolute density. Four concrete formulations were prepared, according to the amount of CCSA (that is at 0 %, 5 %, 10 % and 15 %), in order to carry out tests in the fresh state (slump test, compactness) and in the hardened state, i.e. mechanical tests (compression and bending tests) and physical tests (density test, water absorption, and porosity) after 7, 14, 28 and 60 days. The results of the chemical analyses carried out on the CCSA show that it contains 73.97 % of SiO_2_ + Al_2_O_3_ + Fe_2_O_3_, giving it a pozzolanic character in accordance with ASTM C618 standard. The mechanical behavior at an early age (0–28 days) showed a general decrease in strength for concretes containing CCSA. Replacing 10 % of the cement with CCSA showed the best mechanical performance (compressive and flexural strength) in the long term (60 days) as compared to the control concrete (with 0 % of CCSA). At the same substitution rate the lower absorption and porosity rates were obtained, which are close to those of the control concrete, with relative differences of ±2.43 % and ±3.06 % respectively.

## 1 Introduction

The beginning of the 21st century has been marked by a widespread awareness of the need to limit the impact of human activities on the environment [1]. Activities related to civil engineering account for the largest share of these impacts through the use of ordinary Portland cement (OPC) employed in concrete manufacture, the production of which is estimated at 4.1 billion metric tons in 2023 [[2], [3], [4]], which is expected to reach 4.83 billion metric tons in 2030 [5]. They released high quantities of carbon dioxide (CO_2_) and other greenhouse gas (GHG) emissions into the atmosphere, thus disrupting the balance of nature and causing climate change and global warming by increasing the temperature in the atmosphere during cement production [2,[6], [7], [8], [9], [10], [11]]. In addition, cement production consumes a large amount of natural resources, including limestone and clay. Given the energy-intensive nature of cement production and the global pressure to use sustainable building materials, the immediate replacement of cement with an environmentally friendly material is essential to reduce carbon emissions into the atmosphere [12]. That's why eco-concrete (concrete made from alternative materials such as plant ash and fibers) has been developed to provide all the benefits of an eco-construction material to meet the climatic, environmental, and ecological challenges of any structure [13].

Due to the high cost of building materials and environmental concerns, it is necessary to find alternative building materials that are more economical and sustainable. Thus, the use of vegetable ashes and other residues in concrete is a promising option for reducing construction costs and environmental impacts [[14], [15], [16], [17], [18], [19]]. [20] Vegetable ashes in concrete reduce porosity and improve overall durability [21]. They can also improve the shear strength of concrete by increasing the internal friction between particles, thereby limiting cracking [22]. A number of studies have been carried out on the possibility of replacing cement with vegetable ashes. Palm kernels have been shown to have pozzolanic properties, making them suitable for use as additives to cementitious materials [23]. Incorporating wood ash into concrete has improved mechanical strength in the hardened state [24]. The influence of rice husk ash fineness on concrete properties has been studied [[25], [26], [27]], and the partial replacement (10 %) of cement by rice husk ash has improved absorption and mechanical properties (compression and traction) of hardened concrete [2170]. The results of the properties of corncob ash concrete show that 10 % of corncob ash as a cement substitute is satisfactory in terms of compressive strength [28]. Similar studies have shown that the mechanical performance of concrete with an optimum substitution rate of 20 % was poor at an early age (28 days) compared to the control mix (with 0 % of corncob ash), and that in the long term (120 days), the development of pozzolanic activity in the corncob ash improves the performance of the 20 % substituted concrete, giving it better mechanical properties than the control mix [29]. The partial replacement of cement (at 5 %, 10 % and 15 %) with Egyptian cornstalk ash showed that the physical and mechanical performance of concrete with an optimum substitution rate of 10 % at an early age (28 days) was mediocre compared to the control mix (with 0 % of cornstalk ash). However, in the long term (56 and 90 days), the development of the pozzolanic activity of the CSA improved the performance of the 10 % substituted concrete, giving it better physical and mechanical characteristics than the control mix [30].

In Cameroon, according to AGRISTAT statistics, nearly two million metric ton of corn were harvested in 2022, and almost one million corn stalks were produced [31]. Most of the corn stalks are used as fertilizer or animal bedding and, most importantly, as fuel. Instead of being disposed of as waste. This study aims to determine the applicability of using CCSA as a partial replacement for cement in concrete, which would have direct economic and environmental benefits. The presence of plant matter would also have the effect of reducing the quantity of cement used in concrete and reducing the quantity of plant waste in nature. This study aims to highlight the influence of the rate of cement substitution by CCSA on the properties of fresh and hardened concrete.

**Fig. 1:**
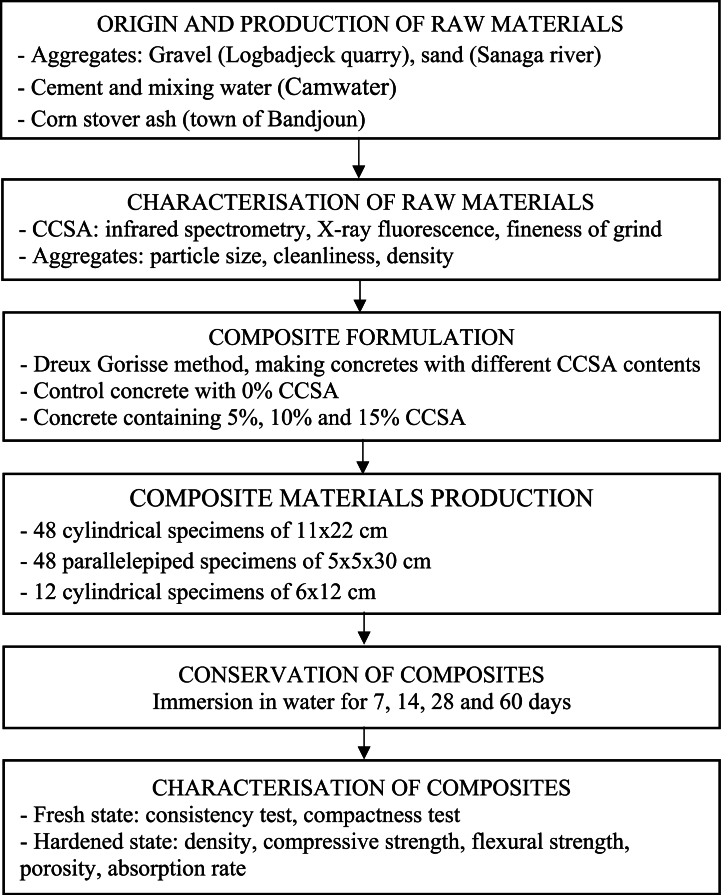
Flow chart of the experimental approach.

In this context, the work carried out here concerns the overall formulation, development and physical and mechanical characterization of an ecological concrete, resulting from the partial substitution (at 5 %, 10 % and 15 %) of artificial Portland cement by CCSA (See Fig. 1). Specifically, at the experimental level, the aim is to identify and characterize the raw materials (particle size, grinding fineness, cleanliness, chemical composition, and density), then to formulate the composites and finally to characterize the physical and mechanical properties of these composites (compactness, consistency, porosity, absorption coefficient, compressive strength and flexural strength), in order to identify the optimum mix.

## 2 Material and methods

### 2.1 Origin and production of raw materials

#### 2.1.1 Cement and mixing water

The cement used to produce concretes was CEM II B-P 42.5 R from the CIMENCAM cement works. The mixing water required to initiate and to maintain the hydration process of the cement and ash was supplied by the Cameroon Water Utilities Corporation (Camwater).

#### 2.1.2 Aggregates: sand and gravel

The sand used has a grain size of 0/5 mm and comes from the Sanaga River in the coastal region of Cameroon. Two types of gravel are used, 5/15 and 15/25 mm, from the Logbadjeck quarry in the coastal region of Cameroon.

#### 2.1.3 Corn stover ash

**Fig. 2:**
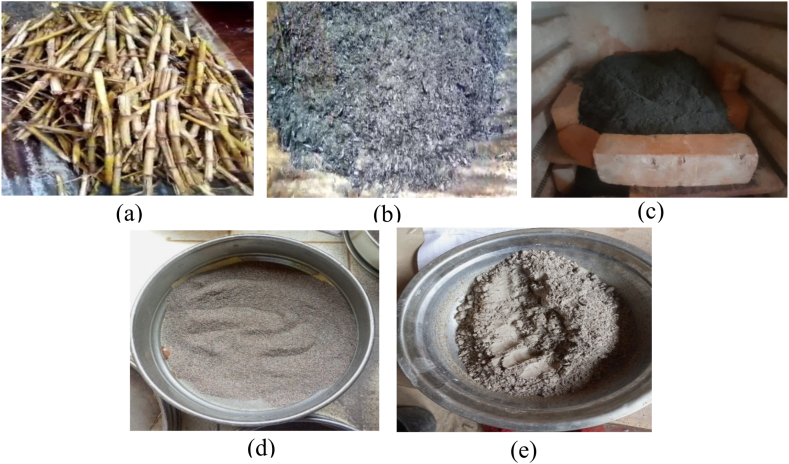
Preparation and production of CCSA: (a) Collection, cleaning and drying of corn stover; (b) Ashes obtained after precalcination; (c) Calcination of ashes in an oven; (d) Sieving with an 80 μm mesh sieve; (e) Final product.

The corn stovers from which the ash is made are harvested in the Koung-khi Division in the West Region of Cameroon, specifically in the town of Bandjoun, one of the areas where corn is widely grown. The corn stovers, as shown in Fig. 2 (a), were harvested in the dry state and then cleaned, left in an open area for 24 h to remove surface moisture before undergoing precalcined without damaging its chemical compounds (Fig. 2 (b)). The precalcined ash was calcined in a controlled oven at 600 °C for 6 h [30] to obtain a homogeneous mixture characterized by particles of similar size and a more active surface. Ashes were then mechanically ground in a mill to obtain the product shown in Fig. 2 (c). The ashes were finally sieved using a standard 80 μm square mesh sieve to remove impurities (see Fig. 2(d)–and (e)).

### 2.2 Characterization of raw materials

#### 2.2.1 Characterization of CCSA

Fourier transform infrared spectrometry (FTIR) was used to identify and characterize the chemical compounds present in the cement and CCSA sample [32]. [33], Chemical analysis by X-ray fluorescence spectrometer was carried out to determine the percentage of different chemical compounds present in the CCSA. The fineness of grinding test was carried out to determine the specific surface area of the CCSA.

**Fig. 3:**
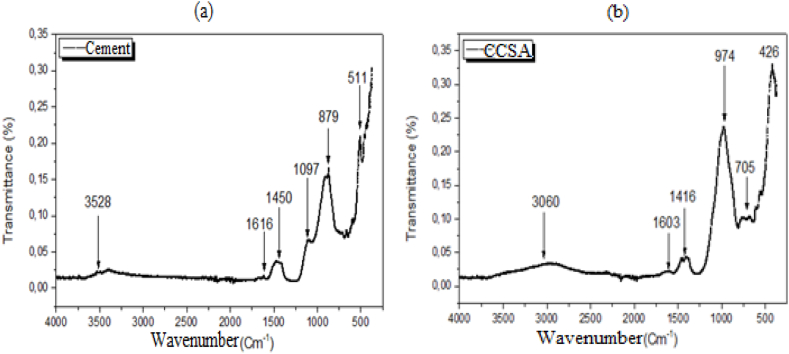
Fourier transformed infrared spectra: (a) cement; (b) CCSA.

The FTIR spectroscopy results presented in Fig. 3 show the absorption bands at different peaks for each vibrational mode. For the O-H bond of Ca(OH)_2_ portlandite, which is a compound formed during cement hydration, a pronounced peak is observed at 3528 cm^−1^ for cement (See Fig. 3 (a)), compared to 3060 cm^−1^ for CCSA (See Fig. 3 (b)). The water molecule in the H-O-H stretching vibration has a peak around 1616 cm^−1^ for cement and 1603 cm^−1^ for CCSA. The CaO molecule associated with the CaCO_3_ molecule shows a peak corresponding to 1450 cm^−1^ for the cement compared to 1416 cm^−1^ for the CCSA. The Si-O-Si and Si-O-Al bonds and the C3S molecules show peaks at 1097, 879 and 511 cm^−1^ for the cement, respectively, compared to 974, 705 and 426 cm^−1^ for the CCSA. These results show the presence in the CCSA, of Si-O-Si, Si-O-Al and O-H bonds present in cementitious materials. In addition, for each bond, the values of the different CCSA peaks approximate those of the cement. These results are consistent with the work of Chowdhury et al., Batt et Garg [34,35] on wood ash and the work of Dewi Sri et al. and Amin et al. [17,26] on rice husk ash.

Table 1 shows the chemical composition results of the CCSA. These results show that the total combined content of silica, alumina and iron oxides (SiO_2_ + Al_2_O_3_ + Fe_2_O_3_) is 73.97 %, which meets the standards defined by ASTM C618.

**Table 1:** Chemical composition of CCSA by X-ray fluorescence spectrometer.

Components	SiO_2_	Al_2_O_3_	Fe_2_O_3_	CaO	MgO	Na_2_O	K_2_O	SO_3_	P_2_O_5_	Loss of ignition
Content (%)	57.38	7.95	8.64	2.83	2.35	0.65	24.79	0.64	0.16	4.9

Blaine's specific surface test gave a value of 5.023 g/cm^2^ for the CCSA sample, an acceptable value according to NF EN 196-6 [36], which recommends a fineness of grind greater than 3.00 g/cm^2^ for cementitious materials. A value of 3100 g/cm^2^ was obtained for CEM II B-P 42.5R from CIMENCAM [37]. Blaine's specific surface area increases with decreasing particle size [38] and the value obtained in this work is higher than those of Salem et al. [30] and Sayoud et al. [39], indicating the very fine nature of our ash. The specific density tests gave values of 3.00 g/cm^3^ and 2.27 g/cm^3^ for the cement and the CCSA, respectively.

#### 2.2.2 Characterization of aggregates

**Fig. 4:**
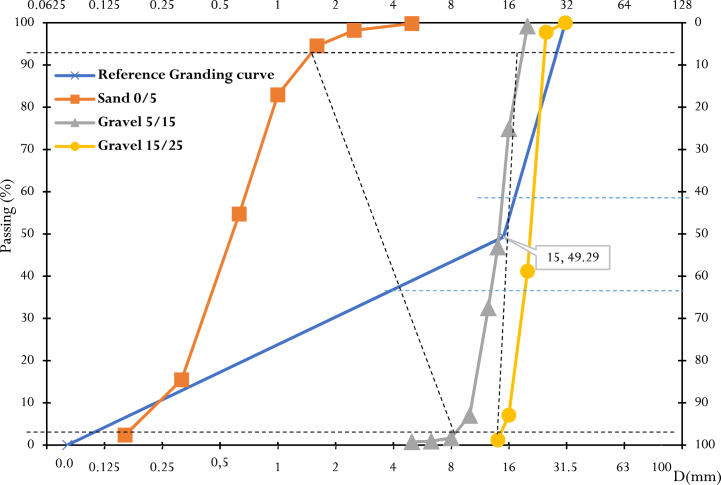
Grading curves for aggregates (sand and gravel).

Sieve analyses were carried out on the 0/5 sand and the 5/15 and 15/25 gravel, following the standard NF EN 933-1. The resulting particle size curves (Fig. 4) show that these respective aggregates have a wide and well graded particle size distribution. The results of the cleanliness tests (Table 2) show that the aggregates are free of impurities and clean for concrete production. The densities of the aggregates are given in Table 2.

**Table 2:** Aggregate characteristics.

Samples	Cleanliness tests (%)	Absolute density (g/cm^3^)	Apparent density (g/cm^3^)	Water content (%)
Sand 0/5	7.32	2.61	1.10	4.77
Gravel 5/15	0.40	2.66	1.48	0.16
Gravel 15/25	0.15	2.75	1.41	0.20

### 2.3 Composite formulation

The concretes were formulated according to the Dreux Gorisse method. The proportions of each aggregate were determined from the reference grading curve and the grading lines (Fig. 4). The proportions of the various constituents required to produce one cubic meter of concrete are given in Table 3, in which four mixes of different compositions were produced by partially replacing cement with CCSA.

**Table 3:** Dosage of constituents in one cubic meter of concrete (kg/m^3^).

Mixtures	Rate of CCSA (%)	CCSA (kg/m^3^)	Cement (kg/m^3^)	Water (kg/m^3^)	W/C	Sand (0/5) (kg/m^3^)	Gravel (5/15) (kg/m^3^)	Gravel (15/25) (kg/m^3^)
M0	0	0	287.50	155.25	0.54	734.71	418.15	804.28
M5	5	14.38	273.12	155.25	0.56	734.71	418.15	804.28
M10	10	28.75	258.75	155.25	0.54	734.71	418.15	804.28
M15	15	43.13	244.37	155.25	0.54	734.71	418.15	804.28

### 2.4 Composite material production

**Fig. 5:**
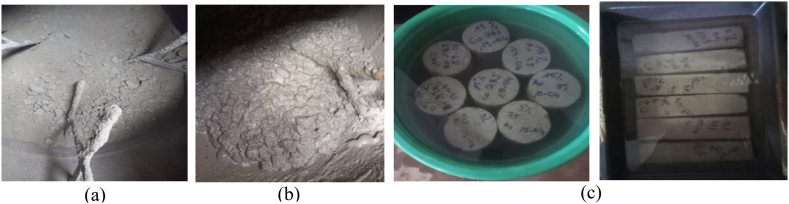
Production of concrete: (a) Mixing of dry constituents (b) Mixing of wet constituents (c) Conservation of composites, curing by prolonged immersion in water.

The procedure for making the concrete is to add the various calculated proportions of the dry components (gravel 15/25, gravel 5/15, sand, cement and CCSA) to the concrete mixer in chronological order and then stir for 2 min consistency (Fig. 5 (a)). Gradually add the water while continuing to stir, stopping when the homogeneous mixture has reached the desired consistency (Fig. 5 (b) and **(c)**). 108 standardized test specimens were made after the fresh concrete was obtained. For the compression tests, 48 cylindrical specimens measuring 11 × 22 cm were made; 48 parallelepiped specimens measuring 5x5x30 cm were made for the flexure test; and 12 cylindrical specimens measuring 6 × 12 cm were made for the absorption and porosity tests. In order to determine the optimum substitution rate, four concretes were formulated, produced and tested with different substitution rates at 0 %, 5 %, 10 % and 15 % by volume of cement, according to the literature [28], [30]. The fresh concrete properties were obtained from consistency tests (Abrams cone slump) and compactness tests after mixing the various constituents. For the hardened concrete properties, 108 standardized specimens were prepared, including 48 cylindrical specimens of ∅11 cm and H22cm for the compression test, 48 parallelepiped specimens of $5\times 5\times 30\;cm^{3}$ for the flexure test, and 12 cylindrical specimens of ∅6cmandH12cm for the absorption and porosity tests. Mechanical compression and flexure tests were performed at 7, 14, 28 and 60 days (3 specimens for each composition). The flow chart in Fig. 1 illustrates the experimental approach.

### 2.5 Conservation of composites

For a minimum of 16 h and a maximum of 3 days, at a temperature of 25 °C ± 5 °C, the hardened concrete specimens are kept in their molds and protected from shock, vibration, and rapid drying. After demolding, the concrete specimens are cured by prolonged immersion in water (7, 14, 28 and 60 days) at a temperature of 20 °C ± 2 °C (Fig. 5 (c)).

### 2.6 Characterization of composites

The properties of the composites are determined in the fresh state (consistency, compactness) and in the hardened state (compressive and flexural strength, absorption rate, porosity). The properties in the fresh state are determined after obtaining the homogeneous concrete paste. Properties in the hardened state are obtained at different concrete ages corresponding to the curing times (7, 14, 28 and 60 days).

#### 2.6.1 Properties of fresh concrete

The compactness of the fresh concrete is determined in accordance with NF P 18–305 standard [40] as shown in the following Eq. (1):

(1) $$c=\frac{\rho_{moy}}{\rho_{\text{theoretical}}}$$

Where $\rho_{\text{theoretical}}$ is its theoretical density (or calculated), while $\rho_{\text{moy}}$ , defined in Eq. (2),

(2) $$\rho_{moy}=\frac{\sum_{i=1}^{n}\left(\frac{m_{2i}-m_{1}}{V}\right)}{N}$$

is its practical (or measured) density. V is the volume of mold, of mass m_1_ and $m_{2i}$ the mass of the mold filled with fresh concrete, while N is the number of test shots.

#### 2.6.2 Properties of hardened concrete

a Average density

The average densities ($\rho_{\text{moy}}$) of the different hardened concretes are determined from the following Eq. (3):

(3) $$\rho_{moy}=\frac{\sum_{i=1}^{n}\left(\frac{m_{i}}{V}\right)}{N}\text{.}$$

b Mechanical testing

**Fig. 6:**
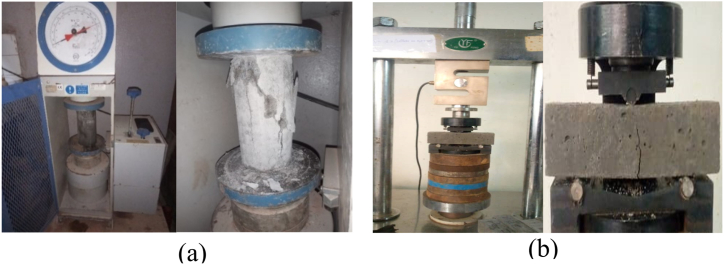
Mechanical testing equipment (a) compression, (b) 3-point bending.

The mechanical tests were carried out at 7, 14, 28 and 60 days from the date of manufacture of the specimens in order to monitor the dynamics of changes in compressive and flexural strength. The compressive and flexural strength tests were carried out in accordance with standards NF EN 12390-4, and NF EN 12390-5 respectively [41,42]. The test arrangements are shown in Fig. 6 (a) and **(b)**. At each age, 3 samples are tested to obtain an average strength value. The relationships used to determine the compressive and flexural strengths are given by Eq. (4).

(4) $$\sigma_{moy}=\frac{\sum_{i=1}^{n}\left(\frac{{4F}_{\max}}{{\pi D}^{2}}\right)}{N},\tau_{moy}=\frac{\sum_{i=1}^{n}\left(\frac{{3F}_{\max}L}{{2b}^{3}}\right)}{N}$$

where: D, b and L are respectively the diameter of the cylindrical specimen, the cross-sectional dimension of the parallelepiped specimen and the length between supports in 3-point bending; $F_{\max}$ is the ultimate load; $\sigma_{moy}$ and $\tau_{moy}$ are the compressive and tensile strengths in 3-point bending, respectively.

c. **Water absorption test** (ASTM D570 Standard)

The absorption test is performed, following the process in Ref. [43], to quantify the rate at which moisture can be absorbed and retained by the pore network of a hardened concrete specimen. To do this, the specimens are dried in an oven for 24h at 105 °C and then placed in a desiccator to cool. Immediately upon cooling the specimens are weighed. The material is then emerged into water, and progressively weighed for 24 h or until equilibrium. The average absorption rate ($\omega_{moy}$) is expressed as a percentage of dry mass and is calculated from Eq. (5).

(5) $$\omega_{moy}=\frac{\sum_{i=1}^{n}\left(\frac{M_{i}\left(t\right)-M_{\text{si}}}{M_{\text{si}}}\times 100\right)}{N}\text{,}$$

in which $M_{i}\left(t\right)$ is the constant wet mass of the test piece after imbibition at time t, $M_{\text{si}}$ the constant dry mass of the specimen after drying in the oven, $M_{i}$ and V respectively the mass and volume of the hardened concrete specimen, t is the weighing time.

d Porosity (NF P18-459, August 2022 Standard)

The most commonly used parameter to describe the microstructure of a porous material is its porosity. Total porosity is the volume fraction of void space between 0 and 1 and provides quantitative information on the volume of voids in the material, but does not provide any information on the size and spatial distribution of these voids. The average porosity ($n_{moy}$) of each hardened concrete specimen is determined from Eq. (6).

(6) $$n_{moy}=\frac{\sum_{i=1}^{n}\left(\frac{M_{3i}-M_{1i}}{M_{3i}-M_{2i}}\times 100\right)}{N}$$

Where $M_{3i}$ is the constant wet mass of the sample weighed in air, $M_{1i}$ is the constant dry mass of the sample weighed in air, $M_{2i}$ is the constant wet mass of the sample measured by hydrostatic weighing.

## 3 Results and discussion

### 3.1 Properties in the fresh state

#### 3.1.1 Compactness of fresh concrete

**Fig. 7:**
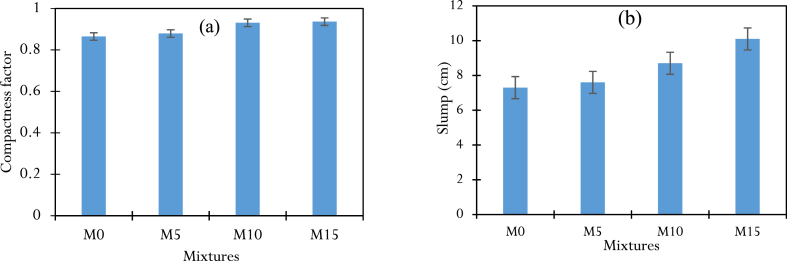
Properties in the fresh state: (a) compactness factors, (b) Abrams cone slumps.

The compactness factors of fresh concrete are illustrated by the histogram in Fig. 7 (a). They increase almost monotonically with the amount of CCSA in the mix. The compactness factors, which vary from 0.86 to 0.94 for CCSA contents of 0 %–15 %, indicate a good distribution of CCSA in the matrix. The increase in compactness with CCSA rate can also be attributed to the ultra-fine ash (fineness of grind 5.023 g/cm^2^), which gives it a high water absorption capacity. These results are in qualitative agreement with the work of Salem et al. and Sayoud et al. [30,39].

#### 3.1.2 Consistency of mixtures

The histogram in Fig. 7 (b) illustrates the cone sag rate of the different mixtures as a function of the rate of substituted rice husk ash. As one can see, the sag increases almost monotonically with the amount of CCSA in the mix. The consistency moves from plastic (M0, M5 and M10) to very plastic (B15). The increase in slump with the rate of CCSA in the mix could be explained by the finer nature of CCSA compared to anhydrous cement, which gives the fresh concrete better workability. Indeed, the very fine particles of CCSA can fill the voids in the cement-aggregate matrix (sand and gravel), thus reducing the internal friction in the matrix and allowing the grains of the solid skeleton to move more easily relative to each other, with fluidity. CCSA-based concrete gives results in line with the requirements of the NF EN 12350-2 standard [44] and in agreement with the work of Mujedu et al. and Mulye et al. [45,46].

### 3.2 Properties in a hardened state

#### 3.2.1 Density

**Fig. 8:**
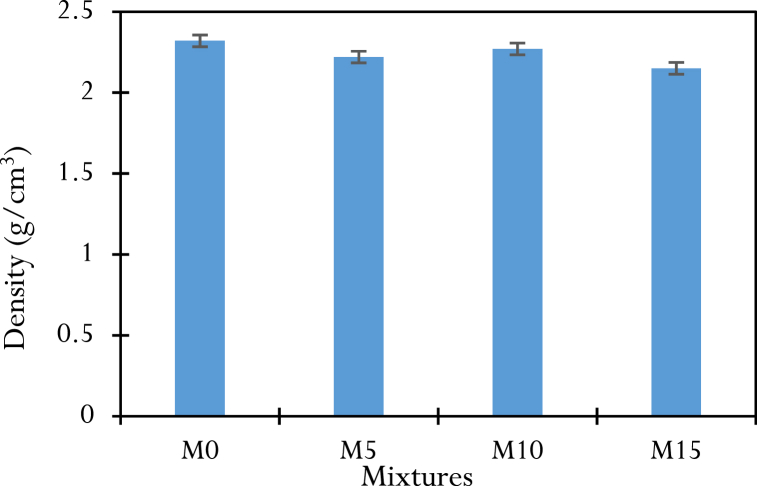
Densities of hardened concrete.

Fig. 8 shows the average densities of the hardened concrete cylinders after 28 days of curing followed by 24 h of drying. The densities in the range of 2.15–2.32 g/cm^3^ correspond to those of traditional concrete. The control mix, with a density of 2.32 g/cm^3^, is denser than the concrete containing the CCSA. This can be explained, on the one hand, by the absolute density of the cement (3.00 g/cm^3^), which is higher than that of the CCSA (2.27 g/cm^3^) and, on the other hand, by the development of the solid C-S-H gel at an early age with the hydration of the cement. The solid C-S-H gel formed when the cement hydrates at an early age closes the capillary pores, making the M0 concrete more compact and dense.

#### 3.2.2 Compressive strength

**Fig. 9:**
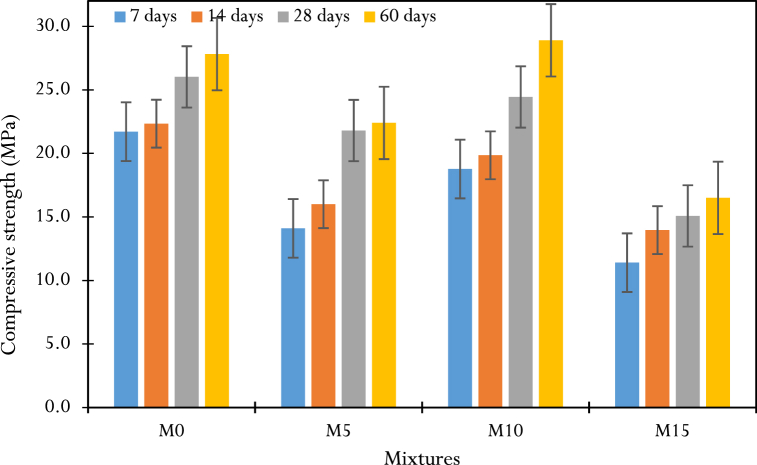
Results of compressive strength at different ages.

The results of compressive strengths are shown in Tables A.1- A.4 in the appendix, which make it possible to draw Fig. 9 for different mixes, which is in qualitative agreement with the work of Salem et al. [30]. The quantitative discrepancies can be explained by the nature of the physico-chemical and mineralogical characteristics of the materials used (cement, ash and aggregates used), the method used to formulate the mixes and the production and conservation of the composites. Several observations are highlighted.

- Compressive strength increases with curing time, regardless of mix design. This is due to the hydration of the cement, which continues over time, making the concrete more compact and resistant.
- M10 with 10 % CCSA has higher compressive strengths than M5 and M15. The lower compressive strength of M15 compared to M10 can be attributed to the reduction of the binder (cement paste), whose pozzolanic activity is optimal at an early age [33,47] and to the increase of CCSA, whose pozzolanic activity leading to the formation of the solid gel C-S-H reaches its optimal value in the long term [30,48]. Conversely, the lower compressive strength of M5 dosed at 5 % compared to M10 is attributed to the low contribution of CCSA present in small amounts [30].
- At an early age (0–28 days), the compressive strength is dominated by the control mix M0, which has higher values than M10 (Fig. 11 (a)); the increase in this strength between 0 and 7 days is greater for M0, which records up to 21.7 MPa at 7 days compared to 18.8 MPa for M10 at the same time. This highlights the slowness of the hydration reaction of CCSA at an early age. Between 7 and 28 days, the kinetics of compressive strength evolution varied for each mixture, but M10 showed a greater rate, with 5.53 % and 18.44 % increase in strength between 7-14 days and 14–28 days, respectively, compared to 2.69 % and 14.23 % for M0 over the same periods. Furthermore, between 28 and 60 days, the increase in compressive strength of M10 was more than 15.57 %, more than twice that of M0, estimated at 6.14 %. This supports the hypothesis of the long reaction time of CCSA (with water), whose pozzolanic activity reaches its optimum state only in the long term.

The long-term increase in strength of concretes with ash replacing cement has been observed by other researchers [30,[49], [50], [51]]. This increase can be attributed to the pozzolanic properties of the CCSA, as shown by X-ray fluorescence spectrometry. In addition, the fineness of the CCSA grind (5.023 g/cm^2^) combined with the high SiO_2_ content (57.38 %) leads to the formation of an additional calcium silicate hydrate (CSH) gel, which contributes to the long-term increase in compressive strength.

#### 3.2.3 Flexural strength

**Fig. 10:**
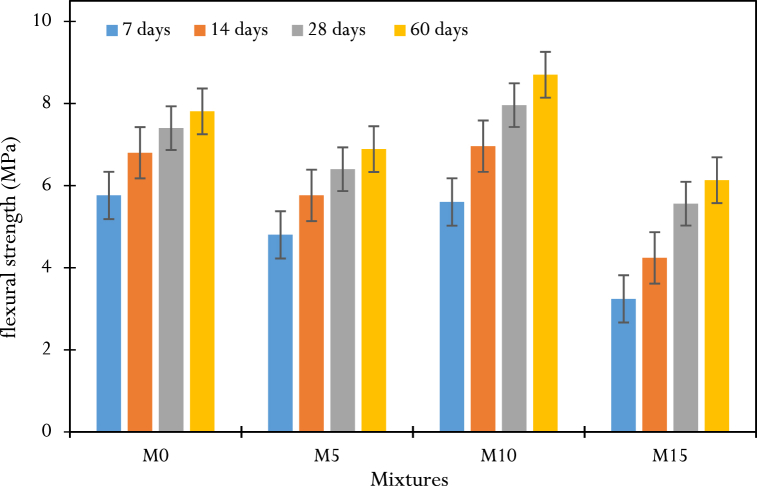
Results of flexural strength at different ages.

The 3-point bending test, carried out on $5\times 5\times 30\;cm^{3}$ prismatic specimens, consisted of applying an increasing load to the center of the specimen, which was simply supported, until it broke, and determining the maximum tensile stress at the level of the lower fiber with the greatest tension. The failure modes of the specimens were similar and were associated with crack opening and propagation in the lower fiber at the point of load application. The test results for each concrete tested are summarized in Tables A.5-A.8 of the appendix, allowing us to draw Fig. 10.

**Fig. 11:**
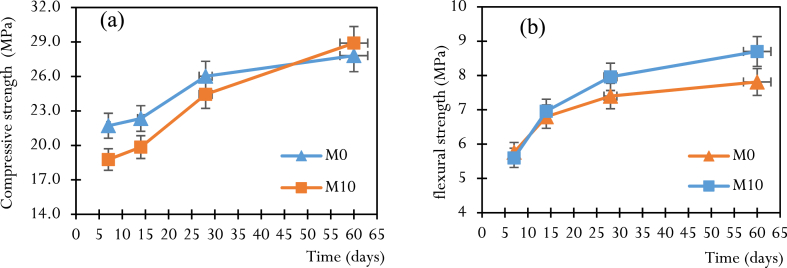
Kinetics of strength changes between M0 and M10: (a) compression, (b) flexion.

Flexural strength increases with curing time regardless of formulation. From 0 to 14 days, the M0 and M10 mixes have almost identical strengths (5.76 and 5.6 MPa at 7 days, respectively, then 6.8 and 6.96 MPa at 14 days) (Fig. 11 (b)). From 14 to 60 days, the evolution of M0 shows a plateau characterized by a small increase in strength (10.88 %) compared to the evolution of M10, which is twice as high (20 %). This result can be attributed to the high grinding fineness of the CCSA present in the M10 mix. At 60 days, M10 shows higher tensile strengths (8.70 MPa) than mixes M5 (6.89 MPa), M15 (6.13 MPa), and control concrete M0 (7.81 MPa). This result is qualitatively in line with the work of Salem et al., Amin et al. and Nigri [30,38,51] and can be explained by the presence in the M10 mix of a sufficient dose of CCSA, necessary for the formation of the additional C-S-H gel resulting from the pozzolanic reaction, which improves the bonding properties and thus limits crack opening.

#### 3.2.4 Rate and kinetics of water absorption

**Fig. 12:**
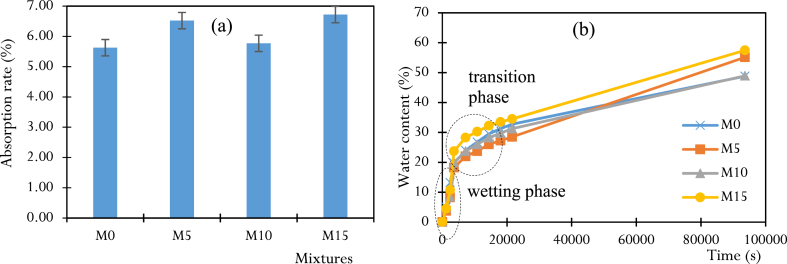
Results of the absorption test: (a) absorption rate, (b) absorption kinetics.

The effect of the rate of partial replacement of cement by CCSA on the absorption properties of concrete is shown in Fig. 12 (a). The tests were carried out on samples that had been cured for 60 days. Regardless of the mix, absorption rates were relatively low. The control mix M0 has the lowest rate (5.63 %) compared to the concrete containing CCSA, M10 (5.77 %), M5 (6.52 %) and M15 (6.72 %). This result is in agreement with the work of Aksogan et al. and Misra et al. [34,52]. The absorption rate and water absorption kinetics of the M10 mix are close to those of the control mix M0 [43] (Fig. 12 (b)).

The water absorption is shown in Table A.9 in the appendix, allowing to draw the kinetics curves shown in Fig. 12 (b) show the same increasing trends in the wetting phase (monomolecular absorption), followed by the transition phase (multimolecular absorption, characterized by the thickening of the water film in the pore network of the samples), which evolves and leads to the plateau characterising a state of water saturation of the samples. In the wetting phase, the absorption kinetics are almost identical for all mixtures, with an average of 5.663 × 10^−3^ g/s (that is 6.646 × 10^−4^ %/s). In the pseudo-saturation phase, the saturation rates are higher for mixtures M5 and M15 (6.52 % and 6.72 % respectively) than for mixtures M0 and M10, which have lower rates (5.63 % and 5.77 % respectively). The resistance of the M10 mix to water migration can be explained by its microstructure (porosity), which is strongly influenced by the optimum pozzolanic activity of this mix, which is responsible for the development of the CSH gel that reduces capillary porosity.

#### 3.2.5 Porosity rate

**Fig. 13:**
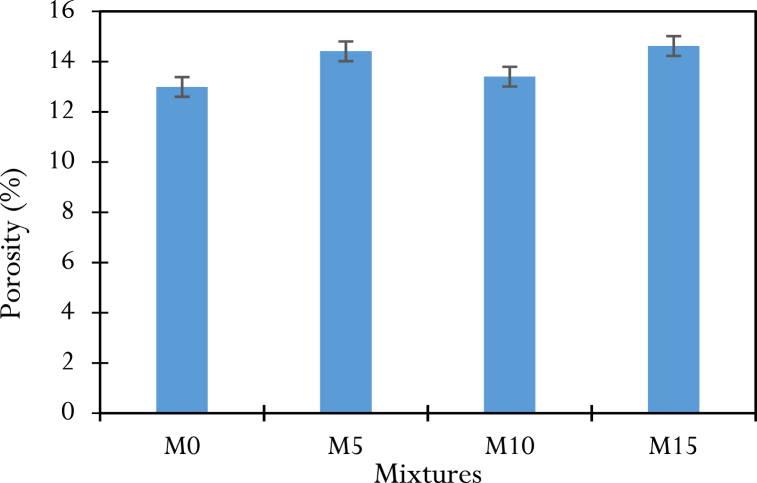
Results of porosity tests.

Porosity is one of the most important microstructural parameters when studying the durability of structures, as its characteristics (pore volume and size) determine the parameters of fluid flow that can occur within the porous medium. Porosity tests were carried out on the various samples after 60 days of curing and the results of porosity are shown in Table A.10, allowing us to draw the curve of Fig. 13. Regardless of the mix, the porosity rates are relatively low, with the control mix M0, having the lowest rate (12.99 %), compared to the concrete containing CCSA, M10 (13.40 %), M5 (14.41 %) and M15 (14.62 %). The low porosity rate of the M10 mix can be attributed to the optimum pozzolanic activity of the high SiO_2_ content in the CCSA (57.38 %), which leads to the formation of a significant CSH gel that helps to reduce the capillary pore volume in the long term.

### 3.3 Significance of the study

Let us remember that the properties of materials in civil engineering are variable and depend on the location [53]. Furthermore, previous studies on corn stalk ash have been carried out in the United States of America and in many other countries, but no study of this type has been carried out either in Cameroon or more particularly on corn stover. Coming from Bandjoun, Western Region of Cameroon. This is why we have placed particular emphasis on the use of Bandjoun corn stover ashes as a substitute for Portland cement which, for us, is new and deserves to be studied. In Fig. 11, Fig. 13 we see that good flexural and compressive strengths are achieved with 10 % substituted ash, along with the low water absorption rate, which should inspire engineers looking to invest in the sector. Civil engineering in Cameroon, for the protection of the environment. Additionally, structural engineers must consider the physical and mechanical properties of 10 % ash concrete when calculating steel reinforcement sections.

## 4 Conclusion

The aim of this research was to investigate the feasibility of producing and using CCSA to make ecological concrete by partially replacing cement with this ash. To achieve this, the CCSA production process was briefly described, as well as the detailed characterization of the materials (physical and chemical properties). From these characteristics, CCSA has shown its applicability as a potential candidate for cement substitution due to its acceptable content of essential pozzolanic compounds found in cementitious materials (SiO_2_ + Al_2_O_3_ + Fe_2_O_3_ in the order of 73.97 %). The CCSA produced, which has a high specific surface area (5.023 g/cm^2^), was used as a cement substitute at three substitution rates (5 %, 10 % and 15 %). The composites were formulated, produced, and characterized, and it was found that the slump and compactness factors increased almost monotonically with the rate of CCSA in the mix. Moreover, the addition of CCSA reduced the compressive strength of the specimens in the early ages. These strengths then increased for a long time (60 days) and even exceeded those of the control concrete. The main results of this work are as follows.

- The replacement of 10 % of the cement with CCSA showed the best mechanical (compressive and flexural strength) and physical (rate and kinetics of water absorption and porosity) performances compared to the other replacement rates (5 and 15 %).
- M10 mix with 10 % CCSA showed decreases in compressive strength at early ages, of −13.36 %, −10.76 % and −6.15 % at 7, 14 and 28 days respectively compared to the control mix M0. In the long period of time (60 days), an increase in strength of +3.77 % was observed for the M10 mix (28.9 MPa) compared to the M0 mix (27.81 MPa).
- In terms of resistance to flexure and cracking, M10 mix containing 10 % CCSA showed a reduction in strength of 2.77 % at 7 days, followed by an overall increase in strength from 2.30 % to 10.25 % between 14 and 60 days.
- Replacing 10 % of the cement with CCSA (M10 mix) showed a lower absorption rate and porosity rate close to those of the control concrete (M0 mix), with relative differences of ±2.43 % and ±3.06 % respectively.
Let us outline that the results found here were based on some specimens tested at each condition, and must be rigorously analyzed, statistically using rigorous tools such as ANOVA, which would certainty explain whether there are differences in some results. The works in this light constitute perspective for future investigations

## CRediT authorship contribution statement

**Martial Ngnihamye Nde:** Writing – original draft, Methodology, Investigation, Formal analysis, Data curation, Conceptualization. **Seidou Youssoufa:** Methodology, Formal analysis, Data curation. **Fabien Kenmogne:** Writing – original draft, Methodology, Formal analysis, Data curation, Conceptualization. **Merveille Meto Kuigne:** Resources, Investigation. **Michel Mbessa:** Validation, Supervision. **Emmanuel Yamb Bell:** Validation, Supervision. **Didier Fokwa:** Validation, Supervision.

## Data and code availability statement

No data was used for the research described in the article.

## Declaration of competing interest

The authors declare that they have no known competing financial interests or personal relationships that could have appeared to influence the work reported in this paper.

## Figures and Tables

**Table A.1:** Results of 7-day compression test

Mixtures	The ultimate compressive strength (kN)			Average (kN)	Compressive stress (Mpa)
	1	2	3		
M0	204	206	209	206,33	21,71
M5	184	179	172	178,33	18,77
M10	138	135	128	133,67	14,07
M15	102	108	115	108,33	11,40

**Table A.2:** Results of 14-day compression test

Mixtures	The ultimate compressive strength (kN)			Average (kN)	Compressive stress (Mpa)
	1	2	3		
M0	212	208	217	212,33	22,34
M5	184	193	189	188,67	19,85
M10	144	159	154	152,33	16,03
M15	129	132	137	132,67	13,96

**Table A.3:** Results of 28-day compression test

Mixtures	The ultimate compressive strength (kN)			Average (kN)	Compressive stress (Mpa)
	1	2	3		
M0	248	242	252	247,33	26,02
M5	232	228	237	232,33	24,44
M10	206	202	210	206,00	21,67
M15	149	138	143	143,33	15,08

**Table A.4:** Results of 60-day compression test

Mixtures	The ultimate compressive strength (kN)			Average (kN)	Compressive stress (Mpa)
	1	2	3		
M0	268	272	262	267,33	27.7
M5	258	248	258	254,67	22.4
M10	206	218	210	211,33	29.8
M15	172	180	162	171,33	16.5

**Table A.5:** Results of 7-day 3-point bending test

Mixtures	Ultimate load in 3-point bending (kN)			Average (kN)	Flexural stress (Mpa)
	1	2	3		
M0	4,2	4,8	5,4	4,80	5,76
M5	4,3	3,8	3,9	4,00	4,8
M10	4,2	4,8	5	4,67	5,6
M15	3	2,3	2,8	2,70	3,24

**Table A.6:** Results of 14-day 3-point bending test

Mixtures	Ultimate load in 3-point bending (kN)			Average (kN)	Flexural stress (Mpa)
	1	2	3		
M0	5,3	5,7	6	5,67	6,8
M5	5,2	4,9	4,3	4,80	5,76
M10	6,01	5,2	6,2	5,80	6964
M15	4,1	3,4	3,1	3,53	4,24

**Table A.7:** Results of 28-day 3-point bending test

Mixtures	Ultimate load in 3-point bending (kN)			Average (kN)	Flexural stress (Mpa)
	1	2	3		
M0	6,5	6,2	5,8	6,17	7,4
M5	5,9	5,3	4,8	5,33	6,4
M10	7,4	6,7	5,8	6,63	7,96
M15	5,2	4,6	4,1	4,63	5,56

**Table A.8:** Results of 60-day 3-point bending test

Mixtures	Ultimate load in 3-point bending (kN)			Average (kN)	Flexural stress (Mpa)
	1	2	3		
M0	6,9	6,9	6,01	6,60	7808
M5	6,4	5,75	5,3	5,82	6,89
M10	8,21	7,5	7,8	7,84	9461
M15	5,5	5,3	5,8	5,53	6,13

**Table A.9:** Results of water absorption test

Mixtures	Constant wet mass (g)	Constant dry mass (g)	Partial absorption rate (%)	Average absorption rate (%)
M0	857,5	810,5	5,79888957	**5,62728112**
	850,5	806,5	5,45567266	
M5	856,5	801,5	6,8621335	**6,52095439**
	850,5	801	6,17977528	
M10	845	798	5,88972431	**5,76972091**
	841,5	796,5	5,64971751	
M15	842	787	6,98856417	**6,72454855**
	847	795,6	6,46053293	

**Table A.10:** Results of the porosity test

Mixtures	constant wet mass weighed in air (g)	constant dry mass weighed in air (g)	constant wet mass measured by hydrostatic weighing (g)	Partial porosity rate (%)	Average porosity rate $n_{moy}$ (%)
M0	857,5	810,5	506,87	13,4044434	**12,9908108**
	850,5	806,5	500,66	12,5771781	
M5	856,5	801,5	494,85	15,2080741	**14,4124657**
	850,5	801	486,98	13,6168574	
M10	845	798	477,45	12,7873759	**12,8266706**
	841,5	796,5	491,74	12,8659652	
M15	842	787	480,6	15,2185944	**14,6234893**
	847	795,6	480,6	14,0283843	
